# Correlation and prediction of gene expression level from amino acid and dipeptide composition of its protein

**DOI:** 10.1186/1471-2105-6-59

**Published:** 2005-03-17

**Authors:** Gajendra PS Raghava, Joon H Han

**Affiliations:** 1 Department of Computer Science and Engineering, Pohang University of Science and Technology, San 31 Hyo-Ja Dong, Pohang 790–784, Republic of Korea; 2 Bioinformatics Centre, Institute of Microbial Technology, Sector 39A, Chandigarh-160036, India

## Abstract

**Background:**

A large number of papers have been published on analysis of microarray data with particular emphasis on normalization of data, detection of differentially expressed genes, clustering of genes and regulatory network. On other hand there are only few studies on relation between expression level and composition of nucleotide/protein sequence, using expression data. There is a need to understand why particular genes/proteins express more in particular conditions. In this study, we analyze 3468 genes of *Saccharomyces cerevisiae *obtained from Holstege et al., (1998) to understand the relationship between expression level and amino acid composition.

**Results:**

We compute the correlation between expression of a gene and amino acid composition of its protein. It was observed that some residues (like Ala, Gly, Arg and Val) have significant positive correlation (r > 0.20) and some other residues (Like Asp, Leu, Asn and Ser) have negative correlation (r < -0.15) with the expression of genes. A significant negative correlation (r = -0.18) was also found between length and gene expression. These observations indicate the relationship between percent composition and gene expression level. Thus, attempts have been made to develop a Support Vector Machine (SVM) based method for predicting the expression level of genes from its protein sequence. In this method the SVM is trained with proteins whose gene expression data is known in a given condition. Then trained SVM is used to predict the gene expression of other proteins of the same organism in the same condition. A correlation coefficient r = 0.70 was obtained between predicted and experimentally determined expression of genes, which improves from r = 0.70 to 0.72 when dipeptide composition was used instead of residue composition. The method was evaluated using 5-fold cross validation test. We also demonstrate that amino acid composition information along with gene expression data can be used for improving the function classification of proteins.

**Conclusion:**

There is a correlation between gene expression and amino acid composition that can be used to predict the expression level of genes up to a certain extent. A web server based on the above strategy has been developed for calculating the correlation between amino acid composition and gene expression and prediction of expression level . This server will allow users to study the evolution from expression data.

## Background

The use of microarray technologies to monitor gene expression in model organisms, cell lines and tissues has become an important part of biological research over the last several years. Even though a number of papers have been published on the analysis of microarray data, particularly on normalization, classification and clustering of data in the last few years [1,2], there is limited work on relation between sequence and expression of gene. In past attempts have been made to establish relation between expression and nucleotide sequence of genes [2-8]. There are studies, which showed the relationship between gene expression and synonymous codon bias [9]. In the past, methods have been developed to predict the expression level of genes from their nucleotide sequences that is based on observation that synonymous codon usage shows an overall bias towards a few codons called major codons [9-11]. Cogan and Wolf 2000 studied the relationship between mRNA concentration and codon bias in detail and found strong correlation (r = 0.62) between codon adaptation index and gene expression [9]. Recently, Jansen et al. 2003 [11] studied the two commonly used numerical indices to measure the expression of genes; i) 'codon adaptation index' (CAI) and ii) 'codon usage' (CU). They improve the performance of two indices using genome wide yeast expression data (15) and achieve correlation r = 0.63 to 0.70 and r = 0.63 to 0.71 of CAI and CU with gene expression level respectively. These studies indicate that it is possible to predict the expression of genes with reasonable accuracy from its nucleotide sequence. There are studies, which indicates directly or indirectly the correlation between amino acid composition and gene expression [6-9,12-14]. The question arises if there is correlation than can we use this knowledge to predict the expression level of genes from amino acid sequence of their protein like nucleotide sequence.

The aim of this study is two fold; to understand the correlation between expression level of genes and primary structure of protein at genome level, and to examine whether the correlation between amino acid composition and gene expression is sufficient enough to derive rules for predicting gene expression from amino acid composition of a protein. A systematic attempt has been made to analyze the gene expression data of *Saccharomyces cerevisiae *(Holstege et al., 1998) to detect the relationship between composition of protein and expression level of gene [15]. We select this data because it was analyzed/used in a number of studies in the past so validation and comparison is easy [9,11-14]. We compute correlation between percent composition and gene expression level, for each residues and observed significant correlation between percent composition and expression level. This means that it is possible to derive rules from proteins whose expression level is known and these rules can be used to predict the expression of other remaining protein in the same organism in the same condition. Similar trend was observed on gene expression data obtained from Jelinsky and Samson, 1999 study [16].

In this study we used a Support Vector Machine (SVM) to learn from known expression data and to predict gene expression of remaining proteins of an organism in the same condition using composition of protein [17-21]. Initially we took amino acid composition as input vector for a protein that has 20 features. Then we tried dipeptide composition as input vector for the SVM where total features are 400. These features provide local order of sequence with composition [18,21]. The method was more accurate when dipetide composition was used as a feature instead of amino acid composition. The performance was nearly same when we tried relative composition and dipeptide composition (in reference to overall composition of genome) instead of absolute composition.

One of the major applications of microarray technology is functional classification of genes where gene expression pattern is used to recognize the functional class of gene[8,10]. It is based on the fact that genes of similar function yield similar gene expression pattern. Brown et al., 2000 developed a SVM based method for predicting five functional classes of genes based on their gene expression in 79 different conditions [19]. We also developed a method based on SVM for recognition of genes belonging to cytoplasmic ribosomes (One of the class used by Brown et al., 2000) using i) gene expression data (79 features); ii) amino acid composition of proteins (20 features) and iii) combination of two. The overall performance in terms of total cost saving [S(M)] was 226, 199 and 234 for gene expression data, amino acid composition and combination respectively. This demonstrates that additional amino acid composition information can improve the performance of functional classification methods based on gene expression data. We also developed a web server that allows one to analysis gene expression data to deduce the relation between expression and composition of residues in protein. This server allows one to train and test the SVM on his or her own gene expression data.

## Results

### Length correlation

**Figure 1:**
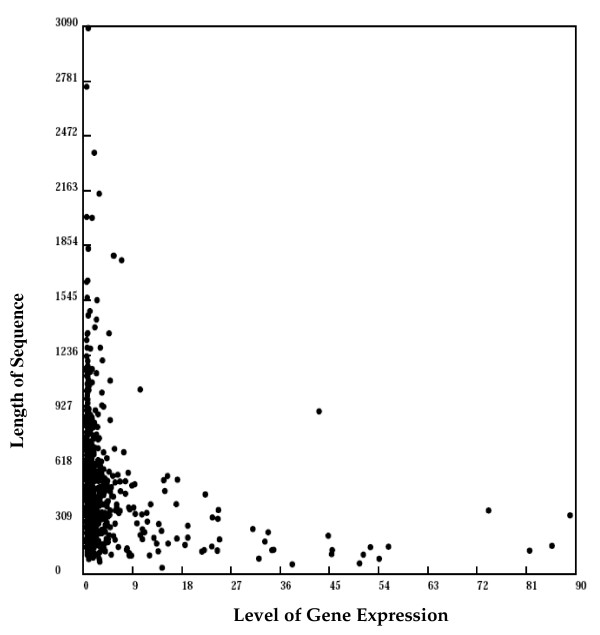
An example plot between expression of genes and length of their protein sequence on one subset of reference data (692 genes). It is generated using LGEpred server option "Standard plot" using option "gene expression on the X-axis".

We examined the correlation between the length of gene and its expression level. A significant negative correlation r = -0.18 was found between the expression and the length of gene. This means that short sequences are expressed more in comparison to long sequences. In order to understand the relationship between expression level and length, we computed the average expression of genes for different length of its protein sequence (Table 1). The average expression is almost inversely proportional to average length of genes. A similar trend was observed on two alternate datasets, where length correlation was r = -0.15 and -0.18 for set1 and set2, respectively. These results agree with previous observations where researchers have shown that metabolic systems prefer to express those genes that are less costly [14,24]. As shown in Table 1, genes having protein length less than 100 amino acids have average expression [e = 15.58]. There was slightly higher expression [e = 2.13] in genes of length more than 1200 in comparison to genes with length in the range of 800–1200. [However, the number of genes was only 168 in this range.] The average expression of genes having up to 200 residues is too high in comparison to long genes. As shown in scatter plot between gene expression and length of protein (Figure 1), most of the genes whose expression is higher than the average are small proteins.

### Correlation between gene expression and protein composition

In the first step, we computed the percent composition of each protein corresponding to genes in our reference dataset (3462 genes) using equation 1. Thus we have 20 values (one for each type of amino acid) for each protein. In the next step, we calculated the correlation between composition of a residue and expression level of gene, for each type of residue. It was observed that some residue types have significant positive correlation, while some others have negative (Table 2). We also computed the correlation for only those genes whose proteins are more than 100 residues in order to see the length effect on correlation. A similar trend was observed except that correlation further improves for residues that have positive correlation and slightly decrease for residues having negative correlation. It is interesting to note that correlation (negative/positive) does not show any relationship with the overall composition of residues in whole genome (Table 2). Following is a brief analysis of both types of residues.

#### Positive correlation

We further analyzed the residues (Ala, Gly, Arg and Val), which showed more than 0.2 positive correlations with gene expression. It is interesting to note that, in general, these residues are less costly for metabolism so they may be preferred for efficient metabolism. We examined whether the average correlation of these positively correlated residues are effective for all the range or it is only in a specified range. For this, we computed the average expression level for genes whose protein has percent composition in different range. As shown in Table 3, the average expression level (E. level) is in increasing order, proportional to percent composition except the range '1–3' where E. level is higher than the next higher range. We examined the proteins, which have percent composition of these residues in range 1–3 and found that most of the corresponding genes are small. As we observed above (See Table 1) that expression level is inversely proportional to the size of gene. Thus, the genes in range '1–3' have unusually high expression for these residues. This is the reason why the correlation between percent composition and gene expression improved further for most of the residues, which have positive correlation when we analyzed only proteins having more than 100 residues (Table 2).

We also computed the average expression level in different range of amino acid composition for those genes whose proteins have more than 100 residues. As shown in Table 4, the average expression level of these residues decreased significantly in the range of 1–3% composition, whereas it was nearly unaffected in higher range of composition. These results show that expression level is proportional to composition of these residues over a wide range.

#### Negative correlation

As shown in table 2, some residues (Asp, Leu, Asn and Ser) have a negative correlation with expression level. The expression data of these residues were further analyzed and the average expression level of genes having different percent composition of these residues is calculated. As shown in table 5, the correlation between percent composition and average expression level is very strong. This shows that the expression for genes of proteins having these residues is not preferred in the cell. In contrast to positively correlated residues, negatively correlated residues showed average expression level as per trend even in the range of '1–3'. It is because lower percent composition is usually found in small genes and both lower percent composition of these residues and short length of proteins are preferred in gene expression. We also computed the average expression level for those genes whose protein have more than 100 residues and found that the average expression level was slightly decreased in lower range (Data not shown).

### Correlation on alternative dataset

#### Dataset 1

As we did with the reference dataset, we computed the correlation between expression level and percent composition on 2693 genes in the alternate Dataset 1. As shown in Table 6, most of the residues, which have positive/negative correlation with gene expression in the reference dataset, also exhibited the same trend in the alternate dataset1. Among positive correlated residues, Arg showed very poor correlation on this dataset, whereas this residue showed high correlation in the reference dataset. We examined this residue and other residues, which have positive a correlation. It is interesting that all these residues including Arg showed increasing average expression level with the range of percent composition.

#### Dataset 2

One of the objectives of this study is to understand the correlation when environment is changed. Here, correlation was computed between gene expression level and percent composition on 2693 genes in alternate dataset 2, after exposure to the alkylating agent methyl methanesulfonate [2]. Overall, similar trend was observed for genes in both alternate dataset (dataset1 & dataset 2) and reference dataset (See table 6). As shown in Table 6, normally, the positiveness (or negativeness) of correlation of a residue was same; only the degree of correlation was different. We examined residues, which have positive correlation. It was interesting that all these residues showed increasing average correlation with the range of percent composition (Table 7).

#### Genes whose expression level changes four fold or more

As shown above, the correlation between gene expression level and percent composition changes slightly in case of untreated and treated genes. The reason is that the expressions of a number of genes are unaffected after treatment. Thus we examined only those 325 genes whose expression level change significantly (> 4-fold). As shown in Table 8, most of residues, which showed high positive or negative correlation in untreated or reference dataset, lose their correlation in treated genes. In other sense, the expression of those genes after treatment increases significantly which favorable residues dominated earlier.

#### Correlation between expression change (EC) and percent composition

One of the major objectives of microarray is to determine the effect on gene expression in different conditions. Thus, we computed the correlation between log(EC) and percent composition, where as EC is (Expression of treated genes)/(Expression of untreated genes). As shown in Table 9, some residues have positive correlation with EC, which means that they increase the expression level in treated case genes. In contrast, some other residues have negative correlation. This is interesting that residues (Ala, Gly and Val) that have positive correlation with expression level of genes have negative correlation with expression changes. In contrast, residues having negative correlation with expression level have positive correlation with expression change. These observations indicate that composition of protein have direct relationship with expression of gene and with the change of expression in different conditions.

### Development of prediction method

The results shown in Table 1 to Table 5 show that there is a strong relationship between primary structure of proteins and expression level of their genes. Based on the above observation, we made a systematic attempt to develop a method for predicting expression level of a gene from its protein sequence; from microarray data of the same organism in a given condition. Based on protein features, we developed two types of prediction methods; one from amino acid composition and the other from dipeptide composition.

#### Amino acid composition

In this case we developed a method using percent composition of proteins as input feature of vector dimension 20 (for 20-residues). A SVM was trained on a training dataset using percent composition as input and gene expression level as output. The SVM was trained using regression mode with linear, polynomial and RBF kernel and achieved correlation coefficient r = 0.46, 0.60 and 0.66 respectively, between predicted and observed values of gene expression, when evaluated using 5-fold cross-validation (Table 10). It is known that SVMs perform better if their input and output values are normalized. As the variation of output (expression level) was very high, we normalize the output. Here, two functions were used to normalize the output values; i) logarithm and ii) square root. The performance of SVM method is shown in Table 10 with these two functions. As shown in Table 10, performance improved significantly when normalized values were used instead of direct value of the expression. The correlation achieved using logarithm and square root functions is r = 0.67 and 0.70 respectively with RBF kernel. The performance of SVM based method with RBF kernel was best when square root was used as the normalization function.

#### Dipeptide composition

We also developed a SVM based method using dipeptide protein feature. The results of this method are shown in table 10. The correlation coefficient r = 0.51, 0.59 and 0.58 were obtained between predicted and observed values of gene expression, when direct, logarithm and square root of gene expression was used as output vector for SVM with a linear kernel. The performance of method was further improved with RBF kernel where correlation reaches to r = 0.66, 0.68 and 0.72 respectively for direct, logarithm and square root respectively. We obtained the best performance at parameters "-c 10 -g 0.01" for RBF kernel in regression mode [23].

#### Membrane and non-membrane proteins

In this study we also tried to develop method for predicting expression level of membrane and non-membrane protein. First we predict membrane proteins in our dataset using popular program SOSUI, which predict 739 membranes, and 2723 non-membrane proteins. We develop SVM based prediction method (RBF kernel with -c 10 -g 0.01, using dipeptide composition) for membrane and non-membrane protein and achieve correlation 0.49 and 0.75 respectively between predicted and actual expression. It is interesting that correlation prediction was too poor for membrane protein, there are two possible reasons one dataset was two small second the amino acid composition of proteins do not exhibit good correlation with gene expression. It was interesting that predictive performance improves from 0.72 to 0.75 for non-membrane proteins despite data set was smaller than original. We also examine the correlation between amino acid composition and gene expression and found that most of residue shows low correlation for membrane and high correlation for non-membrane proteins (See Table 2).

#### Relative composition

In addition to absolute composition (described above) we also tried relative composition. Here input was either relative amino acid or dipeptide composition instead of absolute composition (see Materials and Method). We obtained similar results with relative composition (data not shown).

### Functional classification of genes

First we developed a SVM based method for predicting functional class of genes from their expression data (79 features). We adopted the same strategy as described by Brown et al., 2000 except that we only applied for one class (cytoplasmic ribosomes) instead of five classes. We used the SVM_light package whereas they use their GIST package. The performance of our method in term of TP (true positives), FP (False positives), TN (True Negatives) and cost of saving S(M) [8] on cytoplasmic ribosomes is shown in Table 11. The total cost saving value S(M) of our method was 226 whereas Brown et al. 2000 achieved S(M) value in range of 224 to 229 using various models. The S(M) value achieved by our method was slightly lower than their highest model since they used fine-tuning of parameters and modified the SVM whereas we use the standard SVM with standard RBF kernel. We also developed a method to classify the genes based on their relative amino (See Materials and Methods) acid composition (20 features) and achieved total cost saving value S(M) of 190 (See Table Table 11). It is interesting to note that by using simple amino acid composition one can achieve such a high value which is even better than some models based on gene expression data such as tried by Brown et al., 2002. We achieved total cost saving value S(M) of 234 when we simply combined the output of two SVM methods described above. Here we have not tuned any parameter. We simply add the SVM score of two methods. This clearly indicates that amino acid composition information can play a vital role in improving the performance of classification methods based on gene expression data.

### Web server LGEpred for prediction of gene expression level

Based on the method described in this study, we developed a server that provides various services to the user via Internet.

#### Data analysis

This server allows one to perform various type of analysis on microarray data. This may help users in understanding the relationship between expression of genes and amino acid composition of their proteins. Following is the brief description of options.

**Figure 2:**
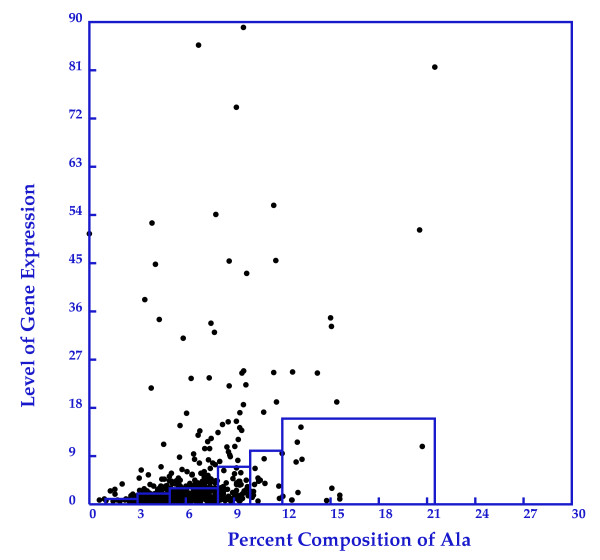
An example plot between expression of genes and percent composition of ALA in their protein. Boxes along the X-axis show the range of composition of ALA and height of the box show the average expression of genes in that range.

• **Correlation coefficient: **This allows the user to compute the correlation between amino acid composition and gene expression from microarray data. The user can generate correlation tables on their microarray data like Table 2 and Table 6.

• **Bin-wise analysis: **One can compute the average expression of genes whose proteins have amino acid composition in a specified range. Basically, it allows comprehensive analysis on binned data. One can generate the average expression tables like Tables 1, 3, 4 &5.

• **Scatter plots of gene expression: **The user can generate scatter plots between gene expression and amino acid composition or length of protein using this option. This allows visualization of relation between gene expression and amino acid sequence on their own expression data. An example figure created using LGEpred server is shown in Figure 1. It provides an option to the user to plot graph by taking expression level on horizontal or vertical axis.

• **Specific plots of gene expression: **The specific plot not only allows us to generate a scatter plot between expression level and amino acid composition but also allows drawing the average expression of genes which have amino acid composition in a specified range (See Figure 2). Using these graphs one can easily detect the relation between expression level and composition in various ranges on their own data.

• **SVM based prediction method**: One of the major features of LGEpred is to allow the users to develop a SVM based prediction method on their own microarray data. This has three major options for the prediction of gene or ORF expression.

• **Training and prediction: **This routine builds a SVM model from users' microarray data using expression level of genes and sequence of proteins. Then it predicts the expression of unknown genes of the same organism in the same condition from their protein sequence using this SVM model.

• **Evaluation and prediction**: This allows users to evaluate the SVM method developed on users' microarray data using LGEpred server. The evaluation is very important in the area of prediction because it provides confidence to the user in using the method of their choice.

• **Prediction from model: **This allows users to predict the expression of genes from their protein sequence using SVM model built using the above options of LGEpred server.

The aim of this server is to provide tools to the users to analyze their own data. All the analyses shown in this manuscript can be performed using LGEpred server. This will allow users to understand their microarray data in depth. This may be used for analyzing cDNA microarray data also where user can provide the expression change instead of expression level in case of oligonucleotide array. This server will also be useful for detecting which residues are preferred in which conditions and why expression of particular genes changes drastically with change of conditions.

## Discussion

Oligonucleotide array is a powerful technique that allows one to study the expression of large number of genes simultaneously [1,2]. Though it is a powerful approach and allows one to study the behavior of genes of an organism in different conditions, it has its own limitations it is expensive, time consuming and has problems in managing and analyzing data. Despite all technical advances it is difficult to study all genes simultaneously of an organism that have a large number of genes like Human Genome. It is also difficult to obtain consistent values in replicates, particularly of those genes whose expression are close to the resolution limits [1,11,17]. In contrast, all the protein sequences of a large number of organisms are available today. The questions arises whether it is possible that we study only limited number of genes [or take those genes from an oligonucleotide array data whose value are consistent in all the samples (duplicate/triplicate)] and use this data to predict the expression level of remaining proteins of the organism in the same condition. This may save a lot of researchers' time and effort in studying whole set of genes of an organism, particularly like human genome. Though there were many studies in the past analyzing the array data, there is no method to predict the expression level of genes. Recently, a paper has been appeared where they describe the procedure to predict the expression of genes [10]. In this paper, they predict the class of genes (genes having the same type of expression behaviors are kept in the same class). They divided the genes into 49 classes and predicted class with 73% accuracy using microarray data from sequences in the 800 bp upstream of genes. From the best of author's knowledge, there is no study, which describes the prediction of gene expression from amino acid sequence of their protein.

This study is the first attempt in this direction to predict the expression level of genes from their protein sequences. In this study we took Holstege et al. 1998 as reference data because it is well studied, and clean [11-15,24-28]. Even though, previous studies indirectly indicate the correlation between amino acid composition and expression level, detailed direct correlations were not shown [3-9]. We studied the correlation between amino acid composition and expression level in detail. Interestingly, some residues showed positive correlation and most of them were small residues. This agrees with the concept of efficient metabolism where researchers demonstrated that proteins having higher composition of less costly amino acids are preferred [12-14]. As shown in Table 5, this correlation trend was shown for whole the range of amino acid composition. The genes having higher range of composition of the positive correlated residues also have higher average expression level. We also observed high correlation between percent composition and expression level for residues Ala, Gly and Val, as previous studies where they found high composition of those residues made of codon GNN in highly expressed genes [14]. There were some exceptions in the case of lowest range, where the average expression level was higher than that of the genes in the next higher range. In fact, most of the genes, which fall in the lowest composition range, belong to category of small genes. As shown in Table 1, the gene expression level has negative correlation with the length of sequence, where smaller genes have higher average expression level (See Table 1 and 2). This is the reason why genes having lower percent composition of positively correlated residues have unusually high expression level. The correlation was increased when we considered only large sequences (> 100 residues). In case of negatively correlated residues trend was more uniform including lower range because low composition of these residues and small sequence are both preferred. Similar trends were observed when we performed our correlation analysis on alternative data sets.

Although the aim of this study is to understand the relationship between expression level and residue composition in normal conditions, we also studied effect on correlation if its conditions are changed. Here, we computed the correlation between expression level and residue composition on alternate dataset 2, which provides expression level of genes when samples are treated with alkylated. Even though the observed correlation between the expression and the composition of the sequence of a specific gene can not be described as a general rule, interestingly, the correlation trend was same; both treated and untreated sets show the same relationship except change in the magnitude of correlation. That is, residues having high positive/negative correlation in dataset 1 showed the same trend in and dataset 2, were same, only magnitude was different. We also analyzed genes whose expression level changes significantly. It is interesting that some residues showed high correlation with fold change of expression. This indicates that in the future it would be possible to predict the gene expression level of proteins in different conditions if we understand in a given condition which residues are favored. These observations suggest more studies in this direction to understand the relationship between gene expression level and primary structure of proteins.

The correlation analysis performed in this study indicates that amino acid composition has correlation with expression. This also indicates that similar sequence will have similar level of gene expression. Now, the question is how to utilize these observations to predict the gene expression of unknown proteins of the same organism. One of the standard practice is to use similarity search tools like BLAST and FASTA for searching similar sequence in dataset of known proteins (whose expression level is known) [29]. The major problems with these tools are that i) they fail in the absence of significant similarity, ii) it is difficult to obtain similarity when the length of query and target sequence are very different, and iii) it is difficult to predict expression level from similarity scores. The machine learning techniques (like ANN and SVM) can be used to learn the relationship between sequence and expression level. The major problem with these techniques is that they cannot be used directly because there are many variations in protein sequence length and these techniques require fixed length patterns. Alternatively, one needs to generate fixed length patterns from these proteins to learn the relationship (or derive the rules from) in known data to predict the gene expression level of other proteins of the same organism. It has been shown in the past that composition, pseudo composition, and dipeptide composition of protein can be used as input pattern of fixed length for classification of proteins using machine-learning techniques [21,31,32]. In this study, first we used the amino acid composition as input and gene expression level as output to develop a SVM based method for predicting gene expression level form amino acid sequence of proteins. As shown in Table 10, we achieved a significant correlation of 0.66 (SVM with RBF kernel) between predicted and observed values of expression level when evaluated using 5-fold cross-validation. It is well known that SVM performs better when its output values are normalized. Here we used two functions (natural logarithm and square root) to normalize expression level(output). These normalization functions, logarithm and square root, increased the correlation from 0.66 to 0.67 and to 0.70 respectively.

It has been observed in past studies of protein classification that accuracy of classification improves significantly when dipeptide composition is used as input instead of single residue (or amino acid) composition [18,21,23]. We also observed similar trends in this study; the correlation between predicted and observed values increased from 0.66, 0.67 and 0.66 to 0.66, 0.68 and 0.72 respectively for without normalization, normalization with logarithm and square root, when dipeptide composition was used as input instead of amino acid composition. This is because dipeptide provides information about sequence order between neighbor residues instead of simple composition. We also tried tripeptide but the results did not improve further because a certain number of patterns were never observed. Our results agree with previous observations where they found that dipeptide is better feature than amino acid composition for SVM based classification [18,21,23].

## Conclusion

The results indicate that there is correlation between expression level and amino acid composition of proteins, which can be exploited to predict the expression level of genes. The correlation between expression level and composition is conditions dependent, which explain the failure of earlier methods of gene prediction based on codon usage and CAI index [11]. In these methods they calculate parameter from expression in one condition and implement for all condition. In our case we proposed condition specific prediction where training and testing is performed in same condition and organism. The expression data is commonly used to classify the genes [8,10]. As far as authors know there is no study, which uses the gene expression and amino acid composition information for classifying or clustering the gene. We made the first attempt in this study and found that combined method performs better than the individual methods (Table 11). We feel that this approach will improve the performance of existing methods in classification and clustering of genes.

The web server LGEpred developed in this study not only allows us to predict gene expression level of proteins of the same organism in the same conditions from its amino acid sequence, but also allows one to understand the relationship between protein sequence and expression level. The server allows the user to compute the following type of correlations; i) correlation between length and expression level; ii) the average expression of genes which has a number of residues of its protein in specific ranges (like 100 to 200); iii) the list of residues which have positive, negative and neutral correlation with expression level; iv) correlation coefficient between residue composition and expression level; and v) the average expression level of each residue when composition is in a specified range. Although the computation level is not very complex or novel, authors feel that it may be very useful for experimental research working in the area of gene expression, because it allows computing the various relations/correlation between protein sequence and expression level from known expression data. This will help the users to detect, which residues are preferred and which are not preferred in their gene expression data, or condition in which they measure the expression. We understand that our method on Holstege data for prediction of expression of genes from protein sequence will only be valid for genes of the same organism in the same conditions. As expression level depends on condition and organism' it is not possible to develop a general method for predicting gene expression. Thus our server allows users to develop their own SVM based method from their known expression data that can be used to predict the expression of genes of the same organism in the same condition. This is a primary study on limited data. In order to understand relationship in depth in various organisms in various conditions, it is needed to analyze all possible available microarray data. In order to assist researchers working with related subjects, we designed LGEpred server by which they can perform various studies on their gene expression data.

## Methods

### Reference expression dataset

In this study, the expression data of Holstege et al. (1998) is used as reference dataset, because its results are obtained from careful averaging of many experiments [1,11,14,17]. All the genes whose expression level is less than 0.5 copies/cell were excluded, because they are close to resolution limits. The final reference dataset contains 3462 genes, whose protein sequences are available in Saccharomyces Genome Database (SGD).

### Alternate dataset

In addition to the reference dataset, we also performed analysis on an alternate dataset obtained from Jelinsky and Samson (1999). In this dataset they examined about 6200 Saccharomyces cerevisiae gene transcript levels with two different environmental conditions. We used 2693 genes whose expression level is more than 0.5 copies/cell in a normal condition and corresponding protein sequence is available in SGD. This dataset consists of two sets; one consists of gene expression data in normal condition and the other after exposure to the alkylating agent methyl methanesulfonate. We also used a subset of 325 genes whose expression level changes significantly when treated with alkylating agent.

### Functional classification of genes

For developing a classification method, we obtained data from Brown et al., 2000 [19]. This data consists of a set of 79-element gene expression vector for 2467 yeast genes. In our study we used 2465 genes whose protein sequence were available in Saccharomyces Genome Database (SGD). Here, we work only one-class protein cytoplasmic ribosomes, which have maximum number of genes 121. Thus our final dataset consists of 121 genes belonging to cytoplamic ribosomes as positive examples and rest 2344 genes as negative examples.

### Five-fold cross validation

The performance of a computational algorithm is often tested by the cross-validation or jackknife method [21,22]. Due to time constraint we evaluate the performance of our method through 5-fold cross validation procedure. In this validation procedure, the dataset was partitioned randomly to 5 equally sized sets. The training and testing of each classifier was carried out five times using one distinct set for testing and rest four for training.

### Amino acid composition

The information of a protein can be encapsulated in a vector of 20 dimensions using amino acid composition of the protein. The composition was used as input in this study, which provides the global information of protein features in the form of fixed length vector. The amino acid composition is the fraction of each amino acid type within a protein. The fractions of all 20 natural amino acids were calculated by using the following equation



where ***comp(i) ***is the fraction of residue or composition of residue of type ***i***. ***Ri ***and ***N ***are the number of residues of type ***i***, and total the number of residue in protein ***i ***(length of protein) respectively. We calculate percent composition by multiplying fraction of residue ***comp(i) ***by 100.

### Dipeptide composition

Dipeptide composition was used to transform the variable length of proteins to fixed length feature vectors. Dipeptide composition has earlier been used by Bhasin and Raghava (2004) for protein classification [18,21]. We adopted the same dipeptide composition based approach in developing SVM method for predicting gene expression level of proteins. The dipeptide composition gave a fixed pattern length of 400 (20×20) possible dipeptides (e.g., Ala-Ala, Aal-Cys, Ala-Asp etc.). The dipeptide composition encapsulates information about the fraction of amino acids as well as their local order. The dipeptide composition was calculated using the following equation.



where ***dpep(i) ***is fraction or composition of dipeptide type ***i***. ***Di ***and ***N ***are the number of dipeptide of type ***i ***and number of residues in protein ***i***, respectively. We calculate percent dipeptide composition by multiplying fraction ***dpep(i) ***by 100.

### Relative composition

The composition (amino acid and dipeptide) described above is absolute composition of proteins. In addition to absolute composition, we also tried relative composition. In this case first, we compute the overall composition (on all proteins of Saccharomyces) each type of residue. Then we compute the relative composition (in reference to overall composition) of each gene using the following equation,





where ***rcomp(i), ocomp(i) ***and ***comp(i) ***are relative, overall and absolute composition of amino acid types ***i ***respectively. Similarly ***rdpep(i), odpep(i) ***and ***dpep(i) ***are relative, overall and absolute dipeptide composition of dipeptide ***i ***respectively.

### Normalization of gene expression level for SVM learning

The gene expression level was normalized to represent on scale of 0 to 10. We used following two functions to rescale the value i) log function, where natural log was used for each gene expression level and ii) sort function, where square root of each expression level was calculated. This normalization is very important in training and testing of SVM for better accuracy.

### SVM training and prediction

In this study, SVM simulation was achieved by using the SVM_light package [20]. This package enables the user to define a number of parameters and to select a choice of inbuilt kernel functions, including Polynomial, RBF, Linear, and Sigmoid. In this study the regression mode of SVM was used.

Let us assume that we have N genes *x*i ∈ R(*i *= 1, 2,..., N) with corresponding target value yi ∈ {target value}(*i *= 1,2,..., N). The *x*i corresponds to the representation of amino acid sequence of the proteins to the SVM. Here, target value is a real value (gene expression level) corresponding to proteins. The dimension of the input vector is 20 for amino acid composition, and 400 for dipeptide composition. The decision function implemented by the SVM can be written as follows:



The value of the α_i _is given by the task of quadratic programming task, maximize subject to 0 ≤ α_i _≤ C, where C is the regulatory parameter controlling the trade off between the margin and training error. Choosing a kernel K for SVM is analogous to the problem of choosing architecture for neural network. In the present work, SVM parameters were all set to default, except the kernel function.

In case of functional classification of genes, input vector consist of 79 features, each feature represents gene expression in one condition. The dataset consists of 121 positive examples (cytoplasmic ribosomes) and 2344 negative examples (non-cytoplasmic ribosomes). The positive examples are very few in comparison to the total data that leads to imbalance in the number of positive and negative training examples. Thus it is difficult for the SVM to correctly classify these genes. In order to handle this problem we replicate the positive examples to match with negative examples during the training of the SVM. Brown et al 2000 modified the SVM to handle this problem.

### Performance measures

The performance of the method has been assessed by computing the correlation coefficient between the actual value of gene expression (experimentally determined) and the predicted value of gene expression [23]. We computed Pearson's correlation coefficient (r), which is the ratio of the covariance between the predicted and experimental values to the product of the standard deviations in the two.



where, X and Y are experimental and predicted value of gene expression respectively. N is the total number of genes in the data set.

## Authors' Contributions

GPSR conceived the project and developed the computer programs for calculating correlation between gene expression and protein sequence composition. GPSR also developed prediction method and wrote the manuscript. JHH coordinated the project, analysis the data and refined the manuscript written by GPSR.

## Figures and Tables

**Table 1:** The average expression level of genes according to the length of protein.

**Length of Protein**	**Total No. of Proteins**	**Average Expression Level (E. Level)**
25–100	59	15.58
100–200	561	8.39
200–400	1168	3.71
400–800	1179	2.51
800–1200	327	1.85
1200	168	2.13

**Table 2:** The correlation between percent composition of residues and gene expression level. The residues which have more than +0.2 correlation are shown in by underline and residue having correlation (negative) -0.15 are shown by bold letter. Second column have percent amino acid compositions in whole yeast genome.

**Amino Acid**	**Overall composition**	**All Genes (3462)**	**Proteins (>100 residues)**	**Membrane Proteins**	**Non-Membrane Proteins**
A	5.49	0.336	0.361	0.401	0.340
C	1.26	-0.003	-0.102	0.030	0.000
**D**	**5.58**	**-0.168**	**-0.152**	**-0.147**	**-0.207**
E	6.54	-0.061	-0.045	-0.105	-0.088
F	4.42	-0.122	-0.109	-0.093	-0.107
G	4.98	0.215	0.217	0.127	0.231
H	2.23	-0.052	-0.056	-0.131	-0.048
I	6.56	-0.136	-0.116	-0.091	-0.128
K	7.35	0.166	0.158	-0.117	0.182
**L**	**9.50**	**-0.208**	**-0.191**	**-0.131**	**-0.212**
M	2.08	-0.087	-0.098	-0.003	-0.094
**N**	**6.17**	**-0.210**	**-0.220**	**-0.209**	**-0.226**
P	4.37	-0.064	-0.057	0.039	-0.086
Q	3.96	-0.052	-0.061	-0.072	-0.065
R	4.43	0.204	0.183	-0.260	0.240
**S**	**8.98**	**-0.152**	**-0.159**	**0.013**	**-0.180**
T	5.92	0.008	0.003	0.279	-0.036
V	5.56	0.269	0.298	0.214	0.294
W	1.04	-0.072	-0.077	-0.043	-0.055
Y	3.38	-0.009	-0.018	-0.030	0.018

**Table 3:** The number of genes and the average expression level of genes which have percent composition of positively correlated residues (e.g. Ala, Gly, Arg & Val) in different bin/range.

Percent	Ala		Gly		Arg		Val	
	
Composition	Genes*	E. Level**	Genes	E. Level	Genes	E. Level	Genes	E. Level
1 – 3	163	2.59	339	2.96	558	3.77	106	2.93
3 – 5	1074	2.11	1180	2.43	1741	2.92	941	2.48
5 – 7	1212	2.80	1193	3.81	802	2.86	1498	3.00
7 – 9	626	5.36	523	6.55	201	8.03	705	5.37
9 – 11	246	9.41	158	7.82	58	20.34	171	13.22
11 – 13	64	15.25	36	12.79	27	17.56	24	16.45
13 – 15	31	15.76	15	12.73	9	29.74	5	22.04
>15	35	15.78	7	13.54	3	23.90	2	19.55

* Total number of genes in this range

** Average expression level of genes in this range

**Table 4:** The number of genes and the average expression level of genes (only genes having more than 100 residues) which have percent composition of positively correlated residues (e.g. Ala, Gly, Arg & Val) in different bin/range.

**Percent Composition**	**Ala**		**Gly**		**Arg**		**Val**	
	
	Genes *	E. Level **	**Genes**	**E. Level**	**Genes**	**E. Level**	**Genes**	**E. Level**
1 – 3	152	2.15	333	2.82	543	3.70	543	3.70
3 – 5	1063	2.05	1166	2.39	1728	2.86	1728	2.86
5 – 7	1204	2.75	1176	3.61	798	2.85	798	2.85
7 – 9	613	4.86	510	6.14	194	7.52	194	7.52
9 – 11	242	9.19	155	7.39	55	19.58	55	19.58
11 – 13	61	15.19	33	12.64	23	17.30	23	17.30
13 – 15	30	15.81	15	12.73	5	29.86	5	29.86
> 15	32	16.52	7	13.54	2	12.85	2	12.85

* Total number of genes in this range

** Average expression level of genes in this range

**Table 5:** The number of genes and the average expression level of negatively correlated residues.

**Percent Composition**	**Asp**		**Leu**		**Asn**		**Ser**	
	
	Genes*	E. Level**	**Genes**	**E. Level**	**Genes**	**E. Level**	**Genes**	**E. Level**
1 – 3	260	9.81	20	14.06	249	8.69	31	12.52
3 – 5	847	3.97	114	12.19	1195	5.21	264	7.85
5 – 7	1505	3.35	467	6.20	1323	2.96	907	5.39
7 – 9	657	3.10	1033	4.05	478	1.72	1202	3.41
9 – 11	117	3.15	1118	3.09	148	1.83	645	2.06
11 – 13	32	2.38	526	2.42	34	3.15	221	2.77
13 – 15	10	2.86	151	1.98	11	0.95	103	1.98
15	5	1.22	32	2.57	9	1.14	87	3.16

* Total number of genes in this range

** Average expression level of genes in this range

**Table 6:** The correlation between percent composition of residues and gene expression level on alternate dataset 1 (untreated) and 2 (treated). The residues in reference dataset having positive correlation are shown by underline and negative correlation by bold letter.

**Amino Acid**	**Alternate Dataset 1 (Untreated)**		**Alternate Dataset 2 (Treated)**	
	
	**All Genes**	**> 100 residues**	**All Genes**	**> 100 residues**
A	0.341	0.348	0.331	0.338
C	-0.052	-0.062	-0.045	-0.051
**D**	**-0.084**	**-0.083**	**-0.055**	**-0.057**
E	0.004	0.016	-0.012	-0.004
F	-0.072	-0.069	-0.057	-0.052
G	0.156	0.165	0.173	0.184
H	-0.075	-0.059	-0.064	-0.051
I	-0.075	-0.085	-0.060	-0.071
K	0.070	0.062	0.017	0.007
**L**	**-0.157**	**-0.155**	**-0.137**	**-0.131**
M	-0.046	-0.057	-0.053	-0.060
**N**	**-0.162**	**-0.156**	**-0.159**	**-0.158**
P	-0.026	-0.035	-0.006	-0.010
Q	-0.037	-0.049	-0.036	-0.047
R	0.018	0.022	-0.046	-0.040
**S**	**-0.102**	**-0.099**	**-0.108**	**-0.109**
T	0.029	0.022	0.041	0.032
V	0.178	0.188	0.193	0.209
W	-0.056	-0.064	-0.034	-0.043
Y	-0.031	-0.040	-0.002	-0.009

**Table 7:** The number of genes and the average expression level of genes which have percent composition of positively correlated residues (e.g. Ala, Gly, Arg & Val) in different bin/range on alternate dataset 1 and 2.

**Percent Composition**	**Ala**			**Gly**			**Arg**			**Val**		
	
	Genes *	E. Level ** (Set 1)	**E. Level (Set 2)**	**Genes**	**E. Level (Set 1)**	**E. Level (Set 2)**	**Genes**	**E. Level (Set 1)**	**E. Level (Set 2)**	**Genes**	**E. Level (Set 1)**	**E. Level (Set 2)**
1 – 3	156	2.51	3.30	248	3.61	4.24	464	4.24	5.44	95	3.50	4.95
3 – 5	784	1.90	2.72	868	2.35	3.09	1317	3.15	4.26	715	2.90	3.59
5 – 7	927	2.44	3.43	965	3.44	4.47	650	2.40	3.27	1173	2.91	3.76
7 – 9	521	3.93	5.46	433	5.81	6.74	157	5.08	4.98	572	4.46	5.39
9 – 11	211	9.90	10.72	122	5.30	5.90	32	12.41	8.29	113	11.02	12.20
11 – 13	47	18.77	14.10	27	9.54	9.55	13	12.03	8.18	16	15.08	16.01
13 – 15	22	16.86	13.19	13	14.68	13.59	3	51.07	32.87	2	22.45	15.25
15	16	26.69	21.81	5	20.46	20.50	2	16.75	15.25	2	36.30	25.65

* Number of genes in this range

** Average expression level of genes in this range

**Table 8:** The analysis of genes in alternate dataset 1 & 2, whose expression changes 4 folds or more when treated with Alkylating agent. Residues showed in reference dataset positive and negative correlation are shown by undeline and bold font respectively.

**Amino Acid**	**Untreated Genes**	**Genes treated with Alkylating agent**
A	0.350	0.265
C	-0.029	0.023
**D**	**-0.099**	**-0.040**
E	-0.070	-0.118
F	-0.087	0.017
G	0.300	0.108
H	0.026	-0.011
I	-0.073	0.067
K	0.075	-0.035
**L**	**-0.200**	**-0.071**
M	-0.041	0.027
**N**	**-0.232**	**-0.165**
P	-0.000	0.028
Q	-0.159	-0.109
R	0.073	-0.137
**S**	**-0.076**	**-0.137**
T	0.002	0.108
V	0.202	0.190
W	-0.131	0.035
y	-0.047	0.043

**Table 9:** The correlation between amino acid composition and log (EC) where EC is (Expression of Treated Genes)/(Expression of Untreated Genes). Residues shows positive and negative correlations are shown by bold font and by underline respectively.

**Amino Acid**	**Correlation with log(Fold change)**
A	-0.184
C	0.055
D	0.029
E	-0.036
**F**	**0.136**
G	-0.118
H	-0.014
**I**	**0.158**
K	-0.111
**L**	**0.220**
M	0.046
**N**	**0.143**
P	0.064
Q	0.068
R	-0.036
S	-0.113
T	-0.110
V	-0.068
**W**	**0.185**
Y	0.076

**Table 10:** The correlation between predicted and experimentally determined gene expression is shown. The value shown by bold font is average correlation on 5 sets of data using in 5-fold cross-validation.

**Normalization Function**	**Linear Kernel**		**RBF Kernel**		**Polynomial Kernel**	
	
	**Amino Acid**	**Dipeptide**	**Amino Acid**	**Dipeptide**	**Amino Acid**	**Dipeptide**
No Function	**0.46 **(0.44, 0.44, 0.48, 0.47, 0.46*)	**0.51 **(0.49, 0.52, 0.51, 0.52, 0.52)	**0.66 **(0.63, 0.60, 0.62, 0.67, 0.75)	**0.66 **(0.63, 0.63, 0.62, 0.66, 0.74)	**0.60 **(0.57, 0.58, 0.60, 0.61, 0.65)	**0.62 **(0.64, 0.62, 0.58, 0.59, 0.66)
**Natural Logarithm**	**0.57 **(0.58, 0.52, 0.59, 0.58, 0.58)	**0.59 **(0.58, 0.55, 0.60, 0.60, 0.60)	**0.67 **(0.65, 0.64, 0.68, 0.68, 0.72)	**0.68 **(0.68, 0.65, 0.65, 0.67, 0.73)	**0.64 **(0.63, 0.61, 0.64, 0.66, 0.67)	**0.66 **(0.67, 0.53, 0.65, 0.68, 0.73)
**Square root**	**0.54 **(0.53, 0.51, 0.56, 0.55, 0.54)	**0.58 **(0.57, 0.56, 0.58, 0.59, 0.60)	**0.70 **(0.69, 0.67, 0.68, 0.71, 0.74)	**0.72 **(0.72, 0.68, 0.67,0.72,0.80)	**0.66 **(0.63, 0.61, 0.64, 0.66, 0.68)	**0.69 **(0.67, 0.53, 0.66, 0.68, 0.73)

* Correlation achieved for each set

**Table 11:** The performance of classification methods on a set of 2465 yeast genes which consists of 121 cytoplasmic ribosomes genes (positive examples) and 2344 other genes (negative examples). GEM and AACM are gene expression based and amino acid composition based SVM methods (RBF kernel) respectively. The SVM parameter for GEM and AACM were "-c 10 -g 0.03" and "-c 10 -g 0.55" respectively

**Method**	**FP**	**FN**	**TP**	**TN**	**S(M)**
GEM	4	6	115	2340	226
AACM	8	22	99	2336	190
GEM + AACM	4	2	119	2340	234
